# Statistical moments of quantum-walk dynamics reveal topological quantum transitions

**DOI:** 10.1038/ncomms11439

**Published:** 2016-04-22

**Authors:** Filippo Cardano, Maria Maffei, Francesco Massa, Bruno Piccirillo, Corrado de Lisio, Giulio De Filippis, Vittorio Cataudella, Enrico Santamato, Lorenzo Marrucci

**Affiliations:** 1 Dipartimento di Fisica, Università di Napoli Federico II, Complesso Universitario di Monte Sant'Angelo, via Cintia, Napoli 80126, Italy; 2 CNR-SPIN, Complesso Universitario di Monte Sant'Angelo, Via Cintia, Napoli 80126, Italy; 3 CNR-ISASI, Via Campi Flegrei 34, Pozzuoli (NA) 80078, Italy

## Abstract

Many phenomena in solid-state physics can be understood in terms of their topological properties. Recently, controlled protocols of quantum walk (QW) are proving to be effective simulators of such phenomena. Here we report the realization of a photonic QW showing both the trivial and the non-trivial topologies associated with chiral symmetry in one-dimensional (1D) periodic systems. We find that the probability distribution moments of the walker position after many steps can be used as direct indicators of the topological quantum transition: while varying a control parameter that defines the system phase, these moments exhibit a slope discontinuity at the transition point. Numerical simulations strongly support the conjecture that these features are general of 1D topological systems. Extending this approach to higher dimensions, different topological classes, and other typologies of quantum phases may offer general instruments for investigating and experimentally detecting quantum transitions in such complex systems.

## Abstract (web summary)

Topological phases play a fundamental role in a variety of physical systems, yet there is a lack of efficient tools for revealing the occurrence of associated quantum transitions. Here, Cardano *et al.* report that such transitions can be identified in the statistics of quantum-walk dynamics and validate this idea in a photonic experimental implementation.

## Graphical abstract

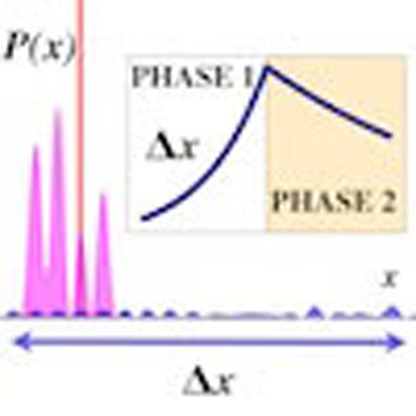

The presence of topological order in matter is responsible for fundamental phenomena, such as the fractional and integer quantum Hall effects^1^,^2^ or the protected surface states observed in topological insulators^3^,^4^. Non-trivial topological phases are related to symmetries and can be characterized by specific topological invariants, defined in terms of the energy band eigenstates. Besides physical systems that naturally exhibit such features, the same topological scenarios have been reproduced in easy-to-access artificial systems (quantum simulators), which represent convenient platforms for the investigation of the intriguing properties associated with these states of matter^5^,^6^,^7^,^8^,^9^. In particular, a variety of simulators based on photonic architectures have been realized^10^,^11^,^12^,^13^,^14^,^15^,^16^.

In the field of quantum simulation of topological phenomena, quantum walks (QWs) are emerging as a versatile tool^17^,^18^,^19^. In its simplest version, a QW is the discrete-time evolution of a particle (the walker) on a one-dimensional (1D) lattice^20^. At each step, the walker can move to either one of the two nearest-neighbour sites of the lattice, as determined by the configuration of an internal two-state quantum system (the coin). Between consecutive steps, the coin state undergoes a rotation that determines the relative probability amplitudes of the subsequent walker move, thus providing the quantum version of the random choice process (the coin toss) characterizing the familiar classical random walk. Among simulators of topological physics, QWs are attracting a wide attention since this simple quantum dynamics can realize all topological phases occurring in one- and two-dimensional systems of non interacting particles^18^; as a first application of this concept, the formation of topologically protected bound states was observed in a photonic QW^10^.

In this paper, we report the study of a QW process that exhibits two distinct topological phases, associated with chiral symmetry in a 1D bipartite lattice. Remarkably, identical symmetries and topological features characterize the Su–Schrieffer–Heeger model (SSH), describing for example the mobile electron dynamics in the poly-acetylene chain^21^, and the effective theory for spin-less 1D superconductors showing *p*-wave pairing^22^. By studying the QW protocol introduced here, we found that the probability distribution moments for the particle position, in the limit of an infinitely long temporal evolution, show a different asymptotic behaviour for the two topological phases: varying an external control parameter, whose value determines the phase of the system, they exhibit a slope discontinuity at the quantum transition. These effects are found to occur also in other 1D topological systems, including models belonging to different topological classes; hence, we conjecture here that they are fully general in 1D. We simulated experimentally such phenomena and proposed a theoretical interpretation based on the dispersion relations and the geometric features of the system eigenstates. Remarkably, our analysis takes into account bulk dynamics only, and not the physical effects manifesting at the edges of the system. With a similar approach topological phases have been recently detected in a non-Hermitian QW,^17^,^11^ while all the models we investigate here are Hermitian.

## Results

### Quantum-walk protocol based on light spin–orbit interaction

In recent years, QWs have been implemented in a variety of physical architectures^23^. In our photonic platform^24^, the walker is encoded in the orbital angular momentum (OAM) of light^25^: discrete positions on the lattice are associated with states |*m*〉, where *m* is an integer, describing a photon carrying *mħ* of OAM along its propagation axis. The coin is encoded in the polarization degree of freedom, that is, in the spin angular momentum (SAM) of photons: vectors |*L*〉 and |*R*〉, representing left and right circular polarizations (SAM of ±ħ per photon), respectively, are the two internal states that determine opposite shift directions in the lattice. The quantum state of the photon after *N* steps of QW is denoted as 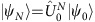, where 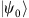 is the initial (input) state and 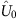 is the unitary evolution operator of a single QW step. In our protocol, this is realized by cascading two optical elements: a quarter-wave plate (QWP) and a *q*-plate (QP). A *q*-plate is essentially a liquid-crystal birefringent cell having the optic axis arranged in a singular pattern^26^, with topological charge *q* (in our case *q*=1/2). This patterned birefringence gives rise to an engineered optical spin–orbit coupling that induces the polarization-controlled shift of OAM. As specified below (see equation 2), besides the charge *q*, the action of this device is determined by the value of the optical retardation *δ*, which can be tuned with an applied electric field^27^; this allows in turn for a fine control of the light spin–orbit interaction mediated by the plate. In our protocol, the QWP is oriented at 90° with respect to the horizontal direction; its action is described by the operator 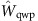, that transforms the polarization states as follows:


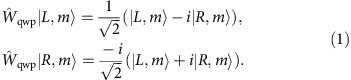


The *q*-plate action is described by the operator 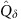, defined as


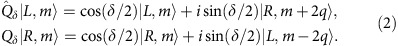


Here we can observe that the spin–orbit interaction introduced by the plate, proportional to sin(*δ*/2), results in an exchange of angular momenta, that is a conversion from SAM to OAM. The combination of 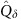 and 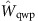 realizes the operator for a single QW step, that is


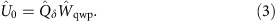


**Figure 1:**
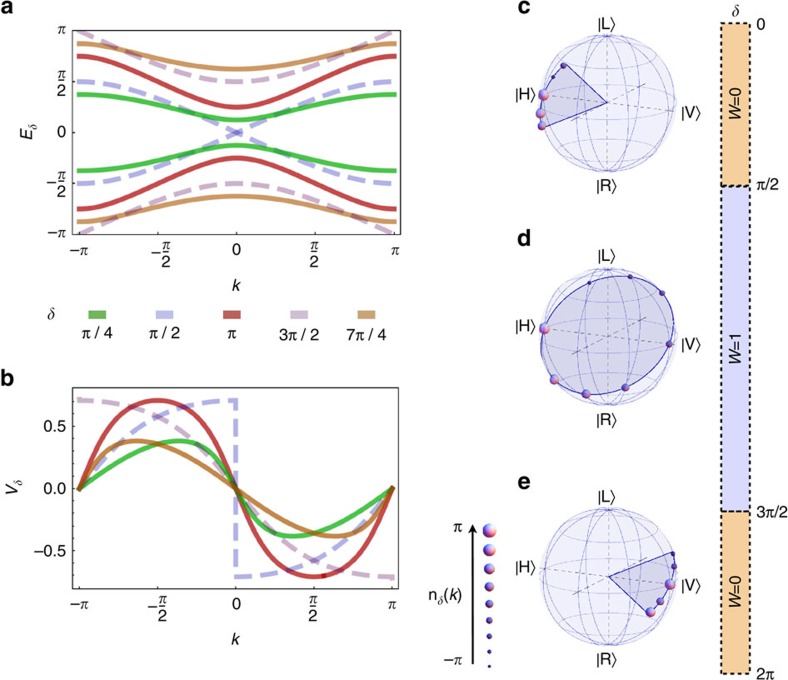
Topological characterization of the QW process. (**a**,**b**) Dispersion relation for the quasi-energy *E*_*δ*_(*k*) and the group velocity *V*_*δ*_(*k*) of the Bloch bands in the QW; for the latter quantity, we plot the lower band only. The dispersion curves depend on the external parameter *δ*; the temporal coordinate is a discrete variable, hence the quasi-energy is defined in a Brillouin zone {−*π*, *π*}. Here we report few examples; colours of the dispersion curves are associated with specific values of *δ*, as shown in the panel legend. Generally, a finite gap separates the energies of the two bands; only at the two points *δ*=*δ*_1_=*π*/2 and *δ*=*δ*_2_=3*π*/2 (blue and purple dashed lines, respectively) the gap vanishes for *k*=0 and *k*=*π*, respectively, causing the presence of a discontinuity in the group velocity (see panel **b**). As shown in **c** points *δ*_1_ and *δ*_2_ represent the boundaries between two topological phases. (**c**–**e**) Topology of the Bloch eigenstates. States |*φ*_*s*_(*k*)〉, representing the polarization (coin) part of the eigenstates of the system, can be represented as points on the surface of a Poincaré sphere, where they are individuated by ±**n**_*δ*_(*k*). Here we represent these states as solid spheres, whose radius is related to the value of *k*, as shown in the legend. As the quasi-momentum *k* spans the Brillouin zone {−*π*, *π*}, chiral symmetry forces these states to lie on a great circle of the sphere. The associated winding number *W* depends on δ, and determines the existence of a non-trivial and a trivial topological phases; the former (*W*=1) occurs when *δ*_1_<*δ*<*δ*_2_, while the latter (*W*=0) is obtained in the remaining part of the interval {0, 2*π*}. As an example, in **c**–**e** we represent the coin eigenstates of a QW with *δ*=*π*/4, *π*, 7*π*/4, respectively. For *δ*=*π* these form a closed loop along the great circle, while in the other cases they go back and forth along a finite arch, whose length depends on the value of *δ* (when *δ*=0 or 2*π*, the arch reduces to a single point).

A topological characterization of QWs with chiral symmetry may be defined in terms of the eigenstates of the operator 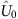 which, due to the lattice translation symmetry, are associated with two quasi-energy bands parameterized by a quasi-momentum *k*, as shown in Fig. 1a. As for momentum *k*, a periodic quasi-energy variable *E* replaces here the usual energy, being the time (= step number) a discrete variable. Accordingly, the dispersion relation for our QW is given by


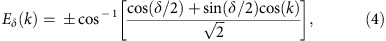


where the two signs correspond to the two bands. As a consequence, the expression of the associated group velocity is


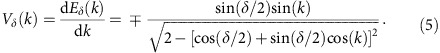


The band eigenstates are given by the product of a walker part, that is plane waves with quasi-momentum *k* in the walker space, and a coin part, denoted as |*φ*_*s*,*δ*_(*k*)〉, where *s*∈{1, 2} is the band index. Conveniently, these states can be represented as points on the Poincaré sphere for light polarization, individuated by a three-dimensional unit vector that we refer to as **n**_*δ*_(*k*); here we report the explicit expressions of its components, that is


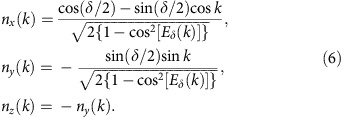


Chiral symmetry constrains vectors **n**_*δ*_(*k*) to lie on a great circle of the sphere. When *k* varies throughout the Brillouin zone {−*π*, *π*}, the number of closed loops (winding number) described by **n**_*δ*_(*k*) is a topological invariant, labelled here as *W*. As shown in Fig. 1c the value of *δ* in the range {0, 2*π*}, that is the strength of the spin–orbit interaction, determines the existence of two phases with a different winding number: a phase with *W*=2*q*=1 occurs when *δ*_1_<*δ*<*δ*_2_, while *W*=0 in the remaining regions. As pointed out in refs 19, 28, for time-periodic systems with chiral symmetry a complete topological classification would require introducing two topological invariants, so *W* alone is not enough. Nevertheless, this is not necessary for the purposes of this work, as we focus our attention on the phase transitions only, while we do not need to identify completely the nature of the topological phases, and it can be shown that all topological phase transitions are associated with a change of *W*^19^,^28^. Therefore, to keep the discussion simpler we will use only the winding number *W* in our discussion. In this sense, we will refer to the phases *W*=0 and 1 as trivial and non-trivial phases, respectively. In Fig. 1a it can be noted that when *δ*=*δ*_1_ and *δ*=*δ*_2_ the two bands are touching and the dispersion is locally linear at *k*=0 and *k*=*π*, respectively, as typically occurs for quantum transitions between topological phases^3^. Interestingly, comparing the expressions of group velocity *V*_*δ*_=d*E*_*δ*_/d*k* (equation 5) and vector **n**_*δ*_(*k*) (equation 6), we can observe that *n*_*z*_=−*n*_*y*_=|*V*_*δ*_|. Thus in the non-trivial phase, where the trajectory of **n**_*δ*_(*k*) is fixed, the maximum of the group velocity is independent of *δ* (see the Supplementary Note 1 and the Supplementary Fig.1 for further details). A recently introduced QW protocol, that is the split-step QW, has very similar properties^10^,^18^.

### Dynamical moments and topological phases

The features of the energy bands and associated eigenstates have profound consequences on the system dynamics, which shows marked differences in the two topological phases^11^,^17^. Here we characterize such different behaviour through the analysis of the moments of the probability distribution *P*(*m*) associated with the walker position, defined as 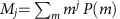. In particular, we consider a photon starting its walk in the position *m*=0 with an arbitrary polarization, that is 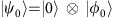, where |*φ*_0_〉 is a generic state in the coin Hilbert space. Simulations of the QW evolution in the large step-number limit show that the moments *M*_*j*_ assume a constant value (independent of *δ*) in the non-trivial phase, and undergo abrupt slope variations at the phase transitions, that is at *δ*={*δ*_1_, *δ*_2_}. In the infinite-steps-limit, we proved that these moments have simple asymptotic expressions in terms of the energy band dispersion relations; in particular, those relative to the first and second moments *M*_1_ and *M*_2_ are the following (see Methods for a proof):


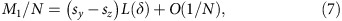



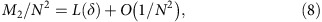


where 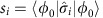, with *i*∈{*x*, *y*, *z*}, are the reduced Stokes parameters, calculated as the expectation values of the Pauli operators 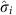 for the coin initial state |*φ*_0_〉. Interestingly, we observe that *M*_2_ is independent of the initial coin state. The quantity *L*(*δ*) appearing in equations 7 and 8 is equal to the square of the group velocity *V*_*δ*_, and hence to the square of *n*_*y*_, averaged over the Brillouin zone:


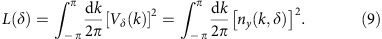


For our QW process, the integral in equation 9 admits a closed form, that is


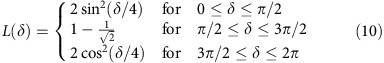


**Figure 2:**
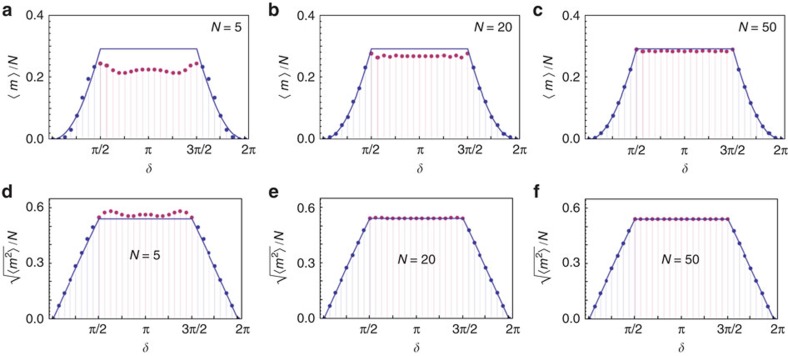
Analysis of statistical moments in a QW. The system, initially localized at *x*=0 with a generic coin state |*φ*_0_〉, undergoes a QW described by the step operator (3). For every plot, purple points are obtained from a numerical simulation, when varying *δ* with steps of *π*/16 in the range {0, 2*π*}; continuous blue lines represent the quantity *L*(*δ*) (**a**–**c**), or 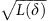 (**d**–**f**) (see equation 10). For the simulation, we prepared the coin in the state {*α*, *β*}={1, 0}, corresponding to {*s*_1_, *s*_2_, *s*_3_}={0, 0, 1}. (**a**–**c**) First-order moment *M*_1_, divided by the number of steps of the walk, as a function of the parameter *δ*, for a walk of 5, 20 and 50 steps, respectively (as specified in each panel). As *N* increases, simulated data converge to the values predicted by equation 7. (**d**–**f**) Square root of the second-order moment, divided by the number of steps *N*. The figures are organized as in **a**–**c**. In this case, we can observe that simulated data converge much faster to the asymptotic values reported in equation 8, with a discontinuity emerging even for a walk of few steps.

**Figure 3:**
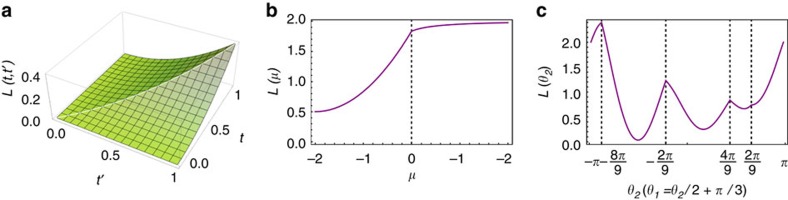
Dynamical moments reveal topological phase transitions in other 1D topological systems. (**a**,**b**) We plot the expected asymptotic values of the second-order moment for a single particle whose evolution is described by the SSH model and the *p*-wave theory for superconductors, respectively, which have the same symmetries as our QW. In the SSH model (**a**), the value of the topological invariant is determined by the parameters (*t*, *t*′). In agreement with our conjecture, non-analyticities of the second-order moment can be observed at the phase change, that is for *t*=*t*′. In the *p*-wave theory, topological features are defined in terms of the chemical potential *μ*. Again, an abrupt slope variation can be appreciated at the transition point *μ*=0. (**c**) Here we consider a QW model having particle–hole symmetry only, in order to show that our results are independent of the specific symmetries characterizing the system. In this model, topological phases are determined in terms of two angles *θ*_1_ and *θ*_2_. These two angles are varied along the trajectory given by the equation *θ*_1_=*θ*_2_/3+*π*/3; similarly to **a**,**b**, the second-order moment after a long temporal evolution shows non-analyticities at the phase changes, identified by vertical dashed lines in the plot. In all simulations, we considered an initial state localized at a specific lattice site. Properties of these models, along with the analysis of other theories, are discussed in detail in the Supplementary Note 2.

In Fig. 2a–c, this expression is plotted as a function of *δ*; as a consequence of the discontinuity present in the group velocity, *L*(*δ*) is a piecewise function, and manifests abrupt slope variations at *δ*_1_=*π*/2 and *δ*_2_=3*π*/2. As shown in Fig. 3 and thoroughly discussed in the Supplementary Note 2 and in the Supplementary Figs 2–6, by applying a similar approach to other 1D topological systems we were able to observe very similar features. For our QW, in the non-trivial phase *L*(*δ*) is constant; this result can be qualitatively understood by looking at how the dispersion law of the group velocity is modified by a change of *δ* (Supplementary Fig. 1). On the other hand, in the trivial phase *L*(*δ*) increases or decreases in the two regions {0, *π*/2} and {3*π*/2, 2*π*}, respectively. Relying on a numerical analysis, we find that such features are robust to the presence of disorder arising from possible experimental imperfections in our setup (details are provided in Methods and in Supplementary Fig. 7). In a QW with a finite number of steps, statistical moments *M*_1_ and *M*_2_ have a continuous behaviour, converging to that given by equations 7 and 8 asymptotically as *N*→∞. For *M*_2_, this convergence is rapid and visible for values of *N* that are small enough to be achieved in an experimental simulation, and it is independent of the coin initial state, whereas for *M*_1_ such process is much slower (see Fig. 2). Thus, as an indicator for identifying the quantum transitions, we chose to use 
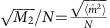
, corresponding to the width of the OAM statistical distribution, normalized to the number of steps. In particular, we implemented a six-step QW and measured the OAM distribution width when tuning the spin–orbit interaction in each *q*-plate, that is *δ*, so as to realize the topological quantum transition.

### Experimental results

**Figure 4:**
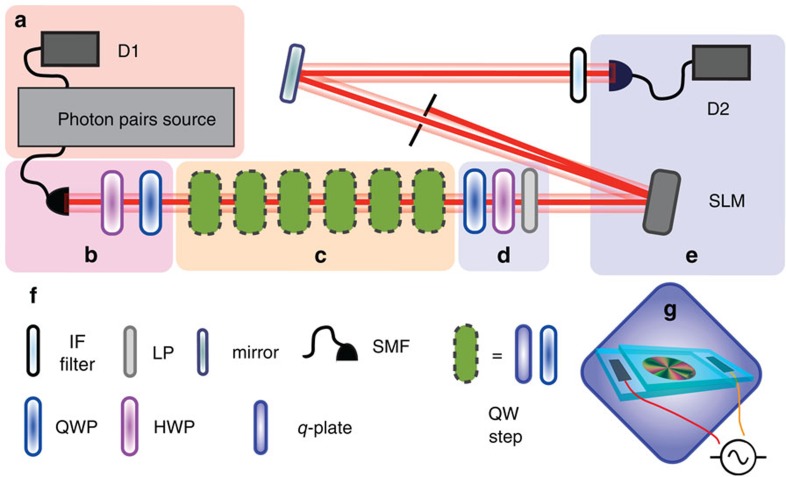
Layout of the experimental setup. (**a**) As described in Methods section, pairs of indistinguishable and temporally correlated photons are generated at the input of the setup. For each pair, they are split by exploiting their orthogonal polarization: one photon is directly sent to an APD D1 and acts as a trigger for the detection of its correlated partner. The latter indeed, after coupling into a single-mode optical fibre (SMF), is sent to the QW setup and finally detected at D2, in coincidence with detection signals from D1. (**b**) The photons undergoing the QW dynamics exits a SMF in the OAM state *m*=0, and then its polarization is prepared in the state |*φ*_0_〉=*α*|*L*〉+*β*|*R*〉; the values of the two complex coefficients *α* and *β* (with |*α*|^2^+|*β*|^2^=1) are set by using a half-wave plate (HWP) and a QWP (apart from an unimportant global phase). (**c**) After the initial state preparation, the photon goes through the six-step QW, with the single step consisting of a QWP oriented at 90° and a *q*-plate. For each *q*-plate, the value of the optical retardation *δ* is controlled by the amplitude of an alternating electric field, induced by an external generator^27^. (**d**) At the exit of the QW, a polarization projection is realized using a second HWP-QWP set followed by an LP. (**e**) The OAM state is then analysed by diffraction on a SLM, followed by coupling into a SMF that is directly connected to the detector D2, consisting in a APD. Before photons are detected, IF centred at 800 nm and with a bandwidth of 3.6 nm are used for spectral cleaning. The latter was required since the photons wavelength strongly affects the action of the devices implementing the QW (*q*-plate and QWP). (**f**) List of all optical elements used in our setup. (**g**) Illustrative picture of a *q*-plate, whose optical retardation is controlled by means of an external oscillating electric field. IF, interference filters; LP, linear polarizer; SLM, spatial light modulator.

**Figure 5:**
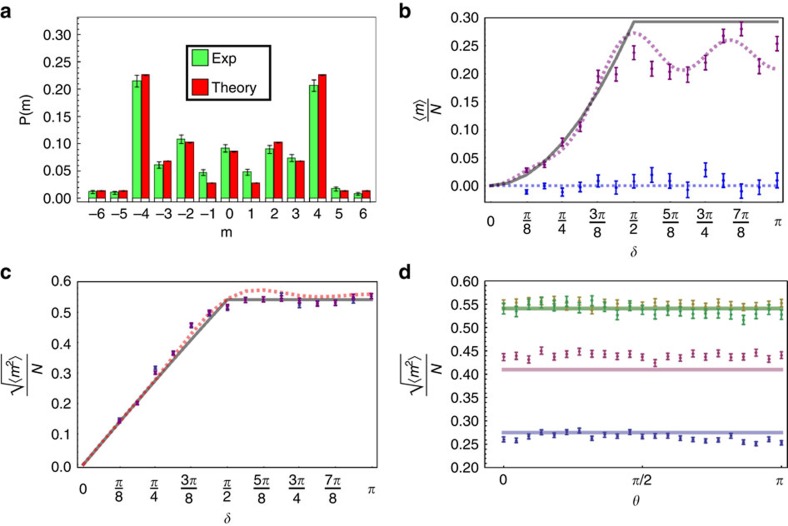
Theoretical predictions and experimental results. (**a**) Example of a probability distribution for the walker: measured (green, left) and expected (red, right) probability distributions after a six-step QW of a photon initially prepared in the state *m*=0 and 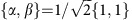, for *δ*=2.95. (**b**,**c**) Measured values of statistical moments *M*_1_/*N* and 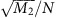, respectively, when varying *δ* in the range {*π*/8, *π*} with steps of *π*/16, for initial states 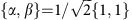 (blue points), and {*α*, *β*}={0, 1} (purple points). Dotted lines represent the corresponding results of numerical simulations. The continuous lines are the asymptotic limits given by equation 8. (**d**) Measured values of 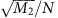 when the initial polarization corresponds to the state cos(*θ*/2)|*L*〉+sin(*θ*/2)|*R*〉, varying the polar angle *θ*∈[0, *π*] with steps of *π*/22; data are collected in correspondence of *δ*=*π*/4 (blue points), *δ*=3*π*/8 (purple points), *δ*=3*π*/4 (yellow points) and *δ*=*π* (green points). For each of these configurations, continuous lines give the corresponding asymptotic limit obtained from equation 8. When *δ*=3*π*/8, experimental data are slightly higher then the associated continuous line; nevertheless, the discrepancy is compatible with the results of numerical simulations for a finite number of steps (see **c**). These data confirm the prediction that *M*_2_ is asymptotically independent of the input coin/polarization state. In all plots (**a**–**d**), the error bars represent statistical errors at 1 s.d., calculated assuming Poissonian fluctuations on single counts.

The layout of the apparatus is shown in Fig. 4. A standard heralded single-photon source is realized at the input of the QW system, as discussed in Methods. Before undergoing the QW evolution, a single photon is prepared in localized initial state (*m*=0), with its polarization (the coin part) described by |*φ*_0_〉=*α*|*L*〉+*β*|*R*〉. The value of the two complex coefficients *α* and *β* (with |*α*|^2^+|*β*|^2^=1) can be tuned by suitably orienting two waveplates that are positioned at input of the setup (see the caption of Fig. 4 for more details). After the QW dynamics, the photon state is analysed in both polarization and OAM so as to determine the output probabilities and the associated moments. Letting *δ* vary in the range {0, *π*} with steps of *π*/16, we determined the corresponding probability distribution of the walker after a six-step QW; a typical example is reported in Fig. 5a. In Fig. 5b,c we plot the measured values for *M*_1_/*N* and 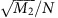, respectively, as a function of *δ*. Experimental data match well the quantum predictions and, as expected, they are close to the asymptotic limit reported in equations 7 and 8 only in the case of 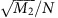. For the latter, the emergence of an abrupt slope variation at *δ*=*π*/2 can be appreciated; the observed non-analyticity is a signature of the underlying quantum transition. In addition, *M*_2_ is observed to be locked to a constant value in the non-trivial phase. On one hand, this result reflects the features of the group velocity and the strong link between the latter and the geometry and topology of the system eigenstates. On the other hand, this is not a general feature of 1D topological systems; in the Supplementary Note 2 we show that for other models, such as the SSH, this plateau can be observed in either of the two phases, depending on the specific trajectory followed in the parameters space. Yet, it is possible to show that for our QW (equation 9) and the SSH model (Supplementary Note 3), the value of *M*_2_ is proportional to the integral of 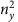 over the Brillouin zone and that the latter always shows a constant value, that is a plateau, when *W*=1. Preparing the initial state of the coin (*m*=0 for the walker) in two non-orthogonal polarizations we also verified that *M*_2_ (unlike *M*_1_) is independent of the coin initial conditions, as expected from equation 8 (see Fig. 5b,c). This aspect was further investigated by measuring 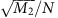 for several initial polarizations, corresponding to specific points along a meridian of the Poincaré sphere, and repeating the experiment for two values of *δ* in each topological sector. The final data, reported in Fig. 5d, match well the predicted results and clearly show that the value of the second-order moment is independent of the coin initial state; this guarantees the robustness of our results with respect to imperfections in the initial state preparation.

## Discussion

The main result of this work is the idea that a signature of quantum transitions between distinct topological phases is present in the behaviour of suitable dynamical observables that are easily accessed experimentally. In the context of 1D topological systems, we propose that the statistical moments of the particle probability distribution in space (in the large step-number limit) provide a convenient choice of such observables. We experimentally validated this proposal by simulating this topological environment within a specific photonic QW architecture. The measured asymptotic moments as a function of a control parameter show slope discontinuities at the phase change, and hence can be used as direct indicators of the phase change occurrence. It is remarkable that these observables reflect bulk properties only, in contrast with the common strategy that relies on investigating topological physics exclusively through edge effects. Numerical simulations show that these features are robust to experimental imperfections and confirm their presence in several other 1D topological systems, thus strongly supporting the conjecture of their general validity in 1D. Moreover, we are currently investigating the possibility to apply similar concepts to 2D systems. Localization induced by spatial disorder is of course expected to affect the final asymptotic behaviour of the dynamics. However, if the disorder is not very strong, it is plausible that an intermediate time regime will still exist, in which the quasi-nonanalytic behaviour of the moments can be detected. In prospect, our approach based on dynamical moments could be applicable to the investigation and to the detection of other classes of quantum transitions, and to topological phases associated with more complex symmetries or with a higher dimensionality, thus helping to shed new light on the physics of topological phenomena and on their simulation in suitable experimental architectures.

## Methods

### Asymptotic expressions of first and second moments

The large step-number limit for the walker distribution moments (equations 7 and 8) can be derived conveniently by evaluating *M*_1_ and *M*_2_ in momentum representation, where they are defined as follows:


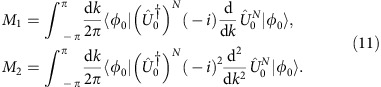


Here *N* is the step-number, 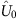 is the single-step evolution operator (3), and |*φ*_0_〉 is the coin initial state. Expanding the evolution operator as


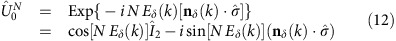


where 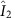 is the identity matrix in 2D, 
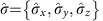
 and the components of **n**_*δ*_(*k*) are those reported in equation 6, it is straightforward to obtain the following equations:


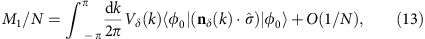



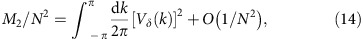


It is important to observe that equations 13 and 14 are valid for any quantum system whose Hilbert space has the same structure as our QW, that is the direct product of an infinite discrete lattice and a two-state quantum degree of freedom. Equation 13, reporting the expression for the first-order moment, can be evaluated considering that *V*_*δ*_=*n*_*z*_=−*n*_*y*_. Applying this substitution, the same equations reads


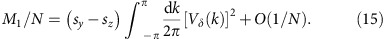


First- and second-order moments reported in equations 14 and 15, respectively, coincide with the equations 7 and 8 of the main text. In the specific case of our QW model, the integral appearing in equations 14 and 15 can be solved analytically. Using the expression for the group velocity reported in equations 5 and 9 yields


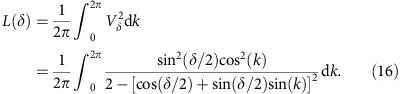


This integral can be calculated by the residue theorem passing to the complex variable *z*=*e*^*ik*^. Then we obtain 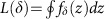, where the integral is along the unit circle in the complex *z*-plane and *f*_*δ*_(*z*) is given by


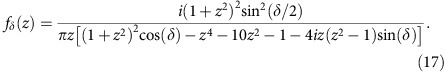


The poles of *f*_*δ*_(*z*) are located on the imaginary axis at 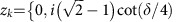, 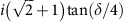, 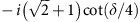 and 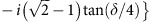 (*k*=1, … 5) and the residues of *f*_*δ*_(*z*) at the poles are given by 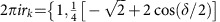, 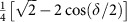, 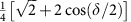 and 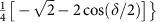, respectively. Apart from the pole at *z*=0, when *δ* varies, the locations of the poles move in the complex plane entering and exiting the unit circle, but only the residues of the poles inside the unit circle contribute to *L*(*δ*). Thus, we find


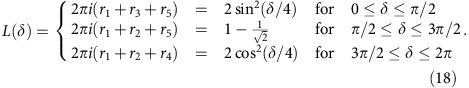


In Supplementary Note 3 and in Supplementary Fig. 6 we show how with the same approach the analytical expression for the function *L* can be found for the SSH model.

### Heralded single-photon source

In the heralded single-photon source (the optical components used to realize this source are not shown in the setup scheme of Fig. 4, for details see ref. 24), laser pulses (100 fs) at 800 nm generated by a titanium–sapphire source (Ti:Sa) with repetition rate 82 MHz shine on a type-I β-barium borate crystal for second harmonic generation; frequency-doubled pulses at 400 nm, with 110 mW average power and linear-horizontal polarization, pump a type-II β-barium borate crystal, cut for collinear and degenerate spontaneous parametric down-conversion. Signal and idler photons, generated in horizontal and vertical linear polarizations, respectively, are spatially separated by means of a polarizing beam-splitter and then coupled into single-mode fibres; the idler photon is directly sent to an avalanche photodiode (APD D1), while the signal one is sent to the QW system. After the QW evolution, such photon is analysed in polarization and OAM and finally detected by APD D2, in coincidence with D1. In this way, the idler photon is used as a trigger, which heralds the presence of the correlated particle passing through the QW setup.

### Experimental imperfections and role of disorder

Experimental realizations of quantum protocols and quantum dynamics exploit specific devices whose behaviour may present deviations with respect to their ideal features. Here we individuate possible imperfections that may arise in our system, discussing in turn how they would affect our QW protocol and, as a consequence, the dynamical moments associated with the walker wavefunction. Interestingly, we find that abrupt variations at the phase change are robust to these kinds of disorder.

A first type of imperfection can result from a bad orientation of the QWPs performing the coin rotation. We considered two different coin input, namely |*H*〉 and |*R*〉 polarizations already used in the experiment, and simulated a 80 steps QW, where at each step a QWP is oriented at ±2° with respect to the correct angle; here we assumed that at 0° a QWP implements the operator reported in equation 1. The ± sign is randomly selected. In Supplementary Fig. 7, we report the results of our numerical analysis. Data clearly show that the second-order moment have no significant differences with respect to the ideal QW; accordingly the phase change can be appreciated still, thus proving the robustness of such indicator. For |*R*〉 as initial state, the mean value has a similar behaviour, whereas something different happens for the |*H*〉 polarization; in the latter case, ideal QW leads to a vanishing mean value. When QWPs have random bad orientations, this is not true anymore, even though deviations from ideal case are smaller than typical experimental errors affecting our data. A second source of errors can arise from the bad alignment of *q*-plates. In particular, their centre could be displaced with respect to the propagation axis of the beam. As a first approximation, the *q*-plate operator reported in equation 2 would be transformed as follows:


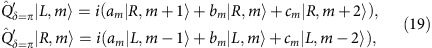


where 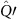 is the operator describing the *q*-plate action when a displacement of its centre is taken into account; here it has been considered the simple case *δ*=*π* (the term proportional to cos(*δ*/2) is the identity operator, which is not affected by such displacement). Moreover, we are explicitly neglecting the coupling to spatial modes of light with different radial distributions, as for instance Laguerre–Gauss modes with radial index *p*≥1. We can note that in equation 19 non-vanishing values for *c*_*m*_ are responsible for next-nearest-neighbour couplings in the OAM lattice, corresponding to an ‘off-diagonal' disorder^29^. Coefficients {*a*_*m*_, *b*_*m*_, *c*_*m*_} can be computed numerically; interestingly, the effects of the *q*-plate displacement are not equal for all OAM states; in particular, deviations from the ideal case becomes negligible for OAM modes with high |*m*|; for such states, *a*_*m*_≃1 and *b*_*m*_≃*c*_*m*_≃0. Similarly to the previous analysis we implemented a numerical simulation of a 80 steps QW for |*H*〉 and |*R*〉 coin input; at each step the *q*-plate is displaced randomly along the *x* or the *y* axis (the *z* axis corresponds to the propagation direction of the light beam). We numerically computed coefficients {*a*_*m*_, *b*_*m*_, *c*_*m*_}, assuming a displacement equal to 5% with respect to the beam transverse dimensions (that is the radius at the waist of the input Gaussian beam). Data reported in Supplementary Fig. 7 show that the effect of such imperfections is negligible. Importantly, the abrupt variation at the phase change can be still appreciated, thus proving the robustness of such features. Even for this case the mean value for |*H*〉 has no significant differences when compared with the ideal case, being it very close to zero for all values of *δ* (within our experimental uncertainties). In conclusion, we want to compare such imperfections with the presence of static disorder, which is typically responsible of localization phenomena. An imperfect orientation of the waveplates leads to a modification of the coin operator that is equal for all OAM states, thus it cannot produce any localization. Effects associated with the *q*-plate displacement are not uniform in the OAM space, but still they are quite different with respect to the typical static disorder, that consists in random site-dependent phase shifts, equal at each step. Moreover, in both cases alterations of the step operator are different at every step of the walk, thus they represent a temporal disorder. Importantly, our simulations show the robustness of moments non-analyticities as indicators of quantum phase changes in such disordered scenarios. Although the spatial static disorder is certainly not significant in our experimental implementation, it can be obviously important for the generalization of our results to other systems. Localization induced by this kind of disorder is expected to affect the final asymptotic behaviour of the dynamics. However, if the disorder is not very strong, it is plausible that an intermediate time regime will still exist in which a quasi-nonanalytic behaviour of the moments can be detected. If ‘localization time' is sufficiently larger than the measurement time, the disorder will not affect the kinks (slightly rounded in real experiments because of the finite measurement time) that signal the occurrence of the topological transitions. Hence, we believe that our main results on the use of moments for detecting topological transitions can be useful also in the context of disordered systems, as long as the disorder is not too strong.

## Additional information

**How to cite this article:** Cardano, F. *et al.* Statistical moments of quantum-walk dynamics reveal topological quantum transitions. *Nat. Commun.* 7:11439 doi: 10.1038/ncomms11439 (2016).

## Supplementary Material

Supplementary Information Supplementary Figures 1-7, Supplementary Notes 1-3 and Supplementary References
