# Supplementary material for: Statistical moments of quantum-walk dynamics reveal topological quantum transitions
Source: Nat Commun. 2016 Apr 22;7:11439. doi: 10.1038/ncomms11439 (PMC4844751; doi:10.1038/ncomms11439)
Supplement: Supplementary Information — Supplementary Figures 1-7, Supplementary Notes 1-3 and Supplementary References [file ncomms11439-s1.pdf]

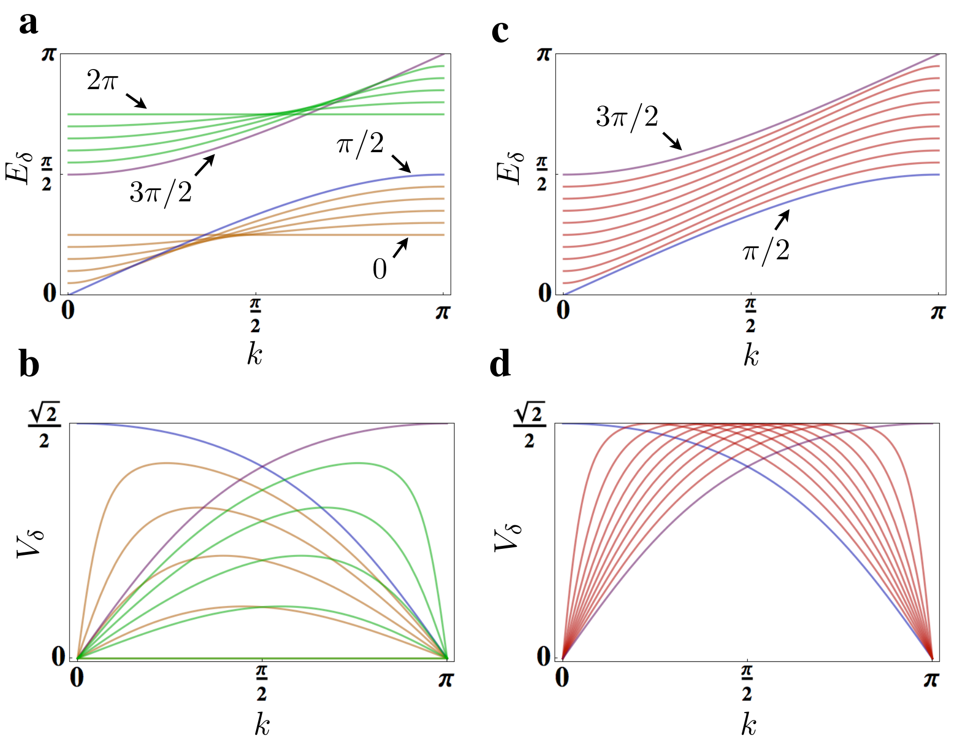

Supplementary Figure 1. The parameter δ and the dispersion relations of our QW system. We plot the dispersion relation for the upper energy band, and the associated group velocity, of a QW system described by the single step operator $\widehat{U}_0$, with varying the external parameter δ in the range $\{0, 2\pi\}$ with steps of $\pi/10$; in these plots, we restrict our attention to half of the Brillouin zone, being the dispersion relation symmetric in the other region $[E(k) = E(-k)]$. In all images, coloured blue and purple curves correspond to the two configurations in which the quantum transition occurs, that is $\delta = \pi/2$ and $\delta = 3\pi/2$, respectively. In panels a-b, coloured orange and green curves correspond to δ being in the intervals $\{0, \pi/2\}$ and $\{3\pi/2, 2\pi\}$, respectively. As a consequence of the energy bands properties, in the former case, as δ increases, the maximum of the group velocity (panel b) becomes larger; in the latter configuration, this behavior is reversed. In panel c-d, coloured red curves correspond to $\delta \in \{\pi/2, 3\pi/2\}$. In this case, the group velocity maximum remains locked to a constant value (panel d), while the corresponding quasi-momentum is drifting, going from $k = 0$ to $k = \pi$ as δ increases. In all panels, the correspondence between values of δ and the associated plots reflects the order in which these curves are displayed.

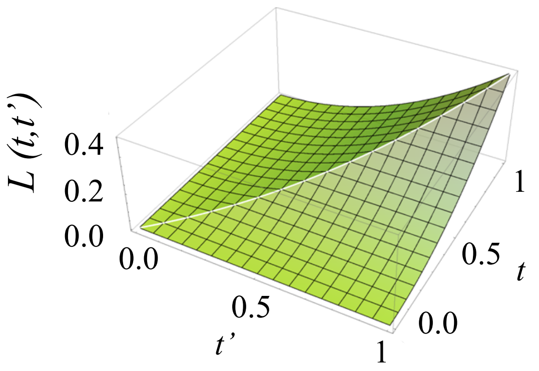

Supplementary Figure 2. Second order moment for the SSH model in the large time limit. We plot the expected asymptotic value of the moment M_2 associated with the probability distribution for a single electron whose dynamics is ruled by the SSH Hamiltonian. As for the QW, we considered a localized initial state. We let the adimensional parameters t and t' vary between 0 and 1.

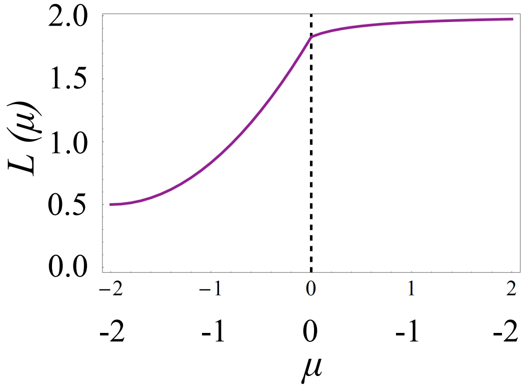

Supplementary Figure 3. Second order moment for the BCS model in the large time limit. We plot the expected asymptotic value of the moment M_2 associated with the probability distribution for a single electron whose dynamics is ruled by the lattice effective Hamiltonian of Bardeen, Cooper and Schrieffer. As for the QW, we considered a localized initial state. We set $\frac{\hbar^2}{m} = 2$ and $\Delta = 1$ in adimensional units. The adimensional chemical potential μ goes from -2 to 2.

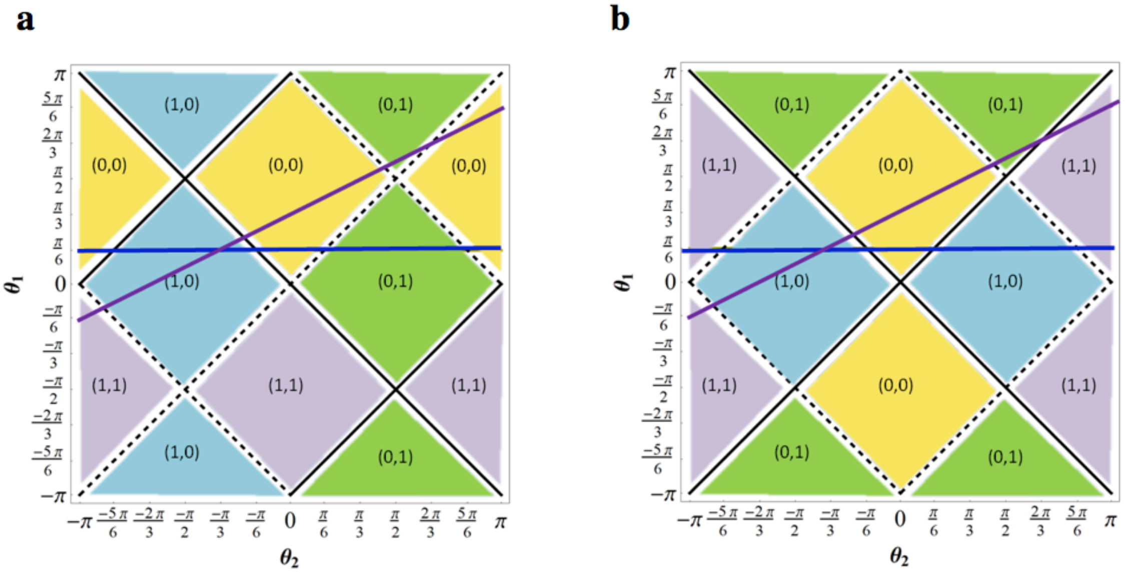

Supplementary Figure 4. Topological maps of the three QWs protocols analyzed in this section. Topological phases for these models are defined by the values of two angles θ_1 and θ_2. In the maps, along continuous lines the energy gap closes in $E = 0$, whereas along the dotted lines it closes at $E = \pi$. Topological sectors corresponding to different values of (Q_0, Q_π) are coloured differently. The blu line is the trajectory obtained varying θ_2, while blocking $\theta_1 = \pi/6$. The purple line represents a different trajectory whose parametric expression is given by $\{\theta_1 = s/2, \theta_2 = s\}$, with $s \in \{-\pi, \pi\}$. The map in panel a applies to the cases of the chiral symmetric and the split-step QW; panel b refers to the particle-hole symmetric QW. For all these three QWs, second order moments have been evaluated for such specific curves in the phase space.

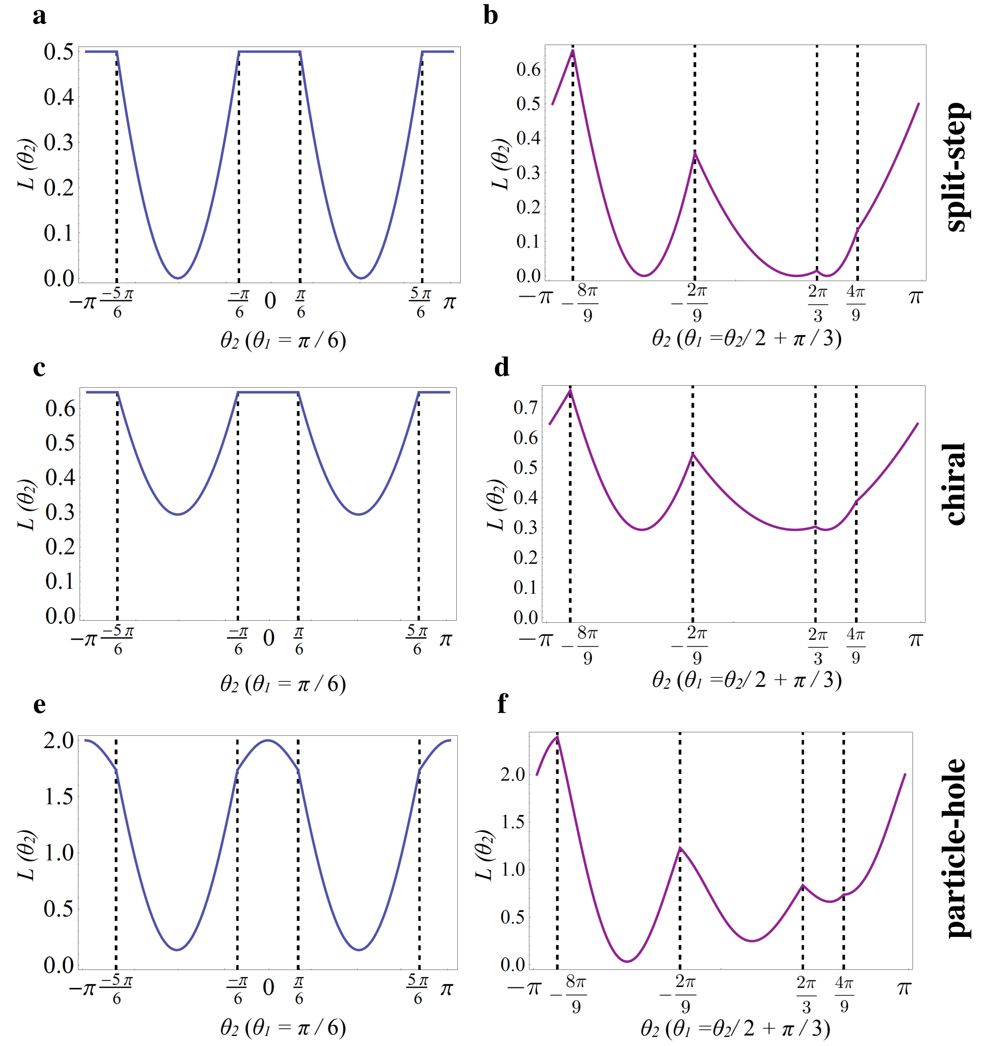

Supplementary Figure 5. Second order moment for the split-step, the chiral symmetric and the particle-hole symmetric QW, respectively, after a large number of steps. We plot the expected asymptotic limit of the moment M_2 corresponding to the probability distribution of a walker starting on a localized initial state, for the split-step evolution [panels a-b], the QW with chiral symmetry only [panels c-d)], and the QW with particle-hole symmetry only [panels e-f)]. (a) In the split-step QW, we set $\theta_1 = \pi/6$. (b) We move on the topological map along the trajectory whose parametric expression is given by $\{\theta_1 = s/2, \theta_2 = s\}$, with $s \in \{-\pi, \pi\}$. Panels c-d and e-f are obtained when considering the chiral symmetric and the particle-hole symmetric QWs, respectively, following the same paths adopted for the plots shown in panels a-b. Independently of the specific models, in all panels we can observe that $\mathcal{L}$ shows slope discontinuities any time the selected paths cross the edges of a topological sector.

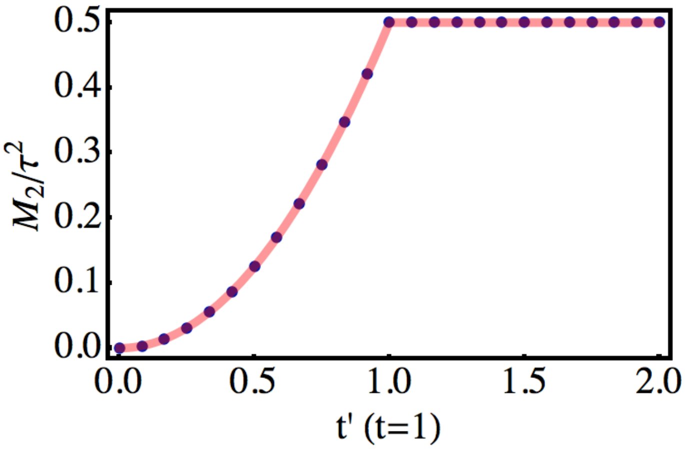

Supplementary Figure 6. Second order moment for the SSH model in the large time limit. We plot the second order moments $\mathcal{M}_2$ corresponding to the probability distribution for a single electron whose dynamics is ruled by the SSH Hamiltonian (3). As for the QW, we considered a localized initial state (at $\tau = 0$). Blue circles represent the data obtained from a numerical simulation, in which we evolved the initial wave function at the time $\tau = 50$ (we recall that we are dealing with adimensional units). In such simulation, we considered t as a constant parameter (i.e. $t = 1$), while varying t' within the interval $\{0, 2\}$ with steps of $2/25$. The red continuous line represents the asymptotic limit calculated using Supplementary Eqs. 32 and 36. We can see that such limit reproduces well the simulated data.

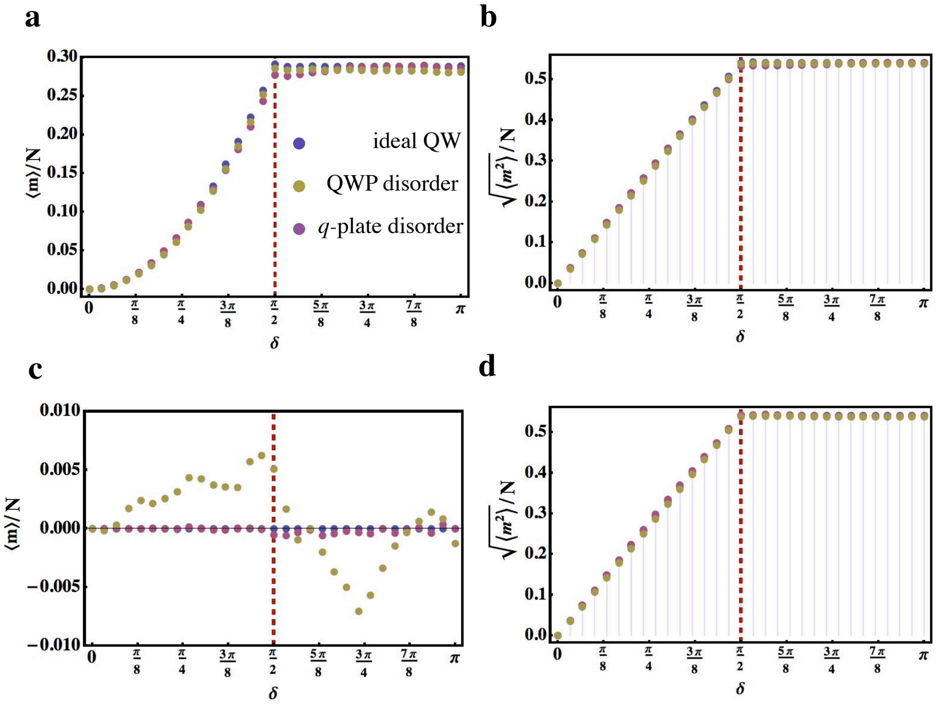

Supplementary Figure 7. Dynamical moments in presence of experimental imperfections. (a-b) First and second order moments obtained from numerical simulations of a 80 steps QW, starting from the initial state $|0, R\rangle$, and varying δ in the range $\{0, \pi\}$, with steps of $\pi/30$. Colored blue, green and purple points correspond to simulations of a QW with no imperfections, with errors in QWP orientation and in q-plate positions, respectively, as shown in the figure legend. A dashed red line at $\delta = \pi/2$ indicates the boundary between the trivial and the non-trivial phases. The presence of both types of disorder does not lead to significant alterations of the moments behavior. The signature of the phase transition, that is the abrupt variation in moments slope, is still observable in both scenarios. (c-d) Numerical results obtained as described for the plots in panels a-b, but considering the state $|0, H\rangle$ as input. Similarly to the previous case, deviations from the ideal case in the mean value behavior are within the experimental errors reported in Fig. 5b. Importantly, for both coin inputs second order moments show the typical discontinuity in their slope at the phase change, thus showing the robustness of such signature with respect to these kinds of perturbations.

Supplementary Note 1. ROLE OF THE EXTERNAL PARAMETER δ IN TAILORING THE STRUCTURE OF THE ENERGY BAND OF THE QW SYSTEM.

In this section, we discuss the dependance of the dispersion relations of our QW system with respect to the value of the parameter δ. The explicit expression of the QW quasi-energies is reported in Eq. 4; we recall that $E_\delta(k)$ are the eigenvalues of the effective Hamiltonian $\hat{H}_{\text{eff}}$; accordingly, they represent a quasi-energy defined in a Brillouin zone $\{-\pi, \pi\}$, as a consequence of the temporal coordinate (the step-number) being a discrete variable. The expression of the related group velocity, that is $V_\delta = \mathrm{d}E_\delta/\mathrm{d}k$, is reported in Eq. 5. In Supplementary Fig. 1, we plot the quasi-energy and the group velocity as a function of the quasi-momentum k, for different values of δ in both the trivial and the non-trivial phases. Here we restrict our attention to the upper band (the energy of the other has only opposite sign), and to half of the Brillouin zone, having the dispersion relation the symmetry $E(k) = E(-k)$. At first glance, it is clear that the energy bands show marked differences for the trivial and non-trivial topological phases. In panel a), we can observe that as δ goes from 0 to $\pi/2$ (or from $3\pi/2$ to 2π), the band covers a larger (smaller) range of energy values; accordingly, in panel c), we can observe that the maximum values for the group velocity increases (decreases). In contrast with what we observed in the trivial phase, in panel b) it can be noted that in the non-trivial phase a change of δ results in a shift of the whole band, plus a tiny deformation of the curve. In panel d), we can observe that this results in a alteration of the group velocity dispersion that keeps constant the associated maximum value; this can be seen as a consequence of the geometric properties of the unit vector $\mathbf{n}_\delta(k)$ in the non-trivial phase, being valid the relation $V_\delta = n_z = -n_y$. The consequences of these features on the statistical moments associated with a QW evolution in the large step-number limit have been discussed in the main text.

Supplementary Note 2. ANALYSIS OF STATISTICAL MOMENTS IN OTHER 1D TOPOLOGICAL SYSTEMS

In this section, we discuss the possible application of our approach to other 1D topological systems, with an internal two-state degree of freedom describing the sublattices features. In

particular, we consider five models characterized by different symmetries and topological invariants; despite their differences, they all show both trivial and non-trivial topologies according to the values of one or more external parameters.

The class of topological phases that can be realized in a system of noninteracting particles is determined by the associated dimensionality and the underlying symmetries of its Hamiltonian. Such symmetries are: the time-reversal symmetry, $\hat{T}\hat{H}\hat{T}^{-1} = \hat{H}$, where the anti-unitary time-reversal operator $\hat{T}$ is defined by $\hat{T} = \hat{U}\hat{K}$, where $\hat{U}$ is a unitary operator and $\hat{K}$ is the complex conjugation operator; the particle-hole symmetry, $\hat{P}\hat{H}\hat{P} = -\hat{H}$, where the anti-unitary particle-hole operator is defined by $\hat{P} = \hat{V}\hat{K}$, where $\hat{V}$ is a unitary operator; and finally the sublattice, or chiral, symmetry $\hat{S}\hat{H}\hat{S}^{-1} = -\hat{H}$ defined by $\hat{T} = \hat{S}\hat{P}$ ($\hat{S}$ is a unitary operator). Systems showing one or all three symmetries can be different in terms of the value assumed by the square of the operators $\hat{T}$ and $\hat{P}$, as each of them can be equal to ± 1. Accordingly, there are five symmetry classes having non-trivial topology. It is straightforward to show that in our quantum walk there is a vector $\boldsymbol{a}$ perpendicular to $\mathbf{n}_\delta(k)$, for all values of k and δ. In the representation where the quantization axis for the coin (z component) is $\boldsymbol{a}$, the operator $\hat{U}$ is the identity and $\hat{V} = \hat{\sigma}_z$. Our quantum walk belongs to the BDI topological class, being $\hat{P}^2 = \hat{T}^2 = 1$ [1–4].

The first three systems analyzed in this section belong to the same topological class as our QW. They are in turn: the Su-Schrieffer-Heeger model for the polyacetylene chain [5], the effective theory for spin-less superconductor showing p-wave pairing [6], and the Kitagawa split-step quantum walk [7]. Then we analyzed two systems that belong to different topological classes: the first possesses only the sublattice or chiral symmetry, while the second one has particle-hole symmetry only. These 1D QW protocols have been proposed in Ref. [8], where Kitagawa et al. have shown that suitably engineered QWs can realize all topological phases in 1D and 2D non interacting systems.

For each model, we report the expression of the Hamiltonian and the associated eigenstates and dispersion relations. Then we evaluate the asymptotic behavior of the second order statistical moment, and plot the associated values computed numerically as a function of the external parameters defining the system topological phases; we show that non-analyticities always occur when the system is driven through a phase change, independently of the specific system. Before discussing the details of each model, we observe that a generic feature of these systems is that, when considering the dynamics of a single particle starting

localized at a given lattice site in a generic internal state (that is in the sublattice degree of freedom), the second order moment can be expressed as

$$\mathcal{M}_2/\tau^2 = \mathcal{L}(\mathbf{a}) + O(1/\tau^2), \tag{1}$$

where components of the vector $\mathbf{a}$ are the external parameters characterizing the system. Here we are considering τ as a continuous or discrete temporal coordinate, depending on the specific system. The quantity $\mathcal{L}$ has the same expression reported in Eq. 9, that is

$$\mathcal{L}(\mathbf{a}) = \int_{-\pi}^{\pi} \frac{\mathrm{d}k}{2\pi} \left[\mathcal{V}_{\mathbf{a}}(k)\right]^2, \tag{2}$$

with the group velocity $\mathcal{V}$ obtained by deriving the quasi-energy of the considered system with respect to k. This result is valid for every model with a two-state sublattice structure, as discussed in the following; hence, for each case, we will evaluate the asymptotic behavior of second order moments by computing numerically the integral 2. In Supplementary Note 3 we also compare these simulations with the results obtained when the second order moments are extracted directly from the final wave-function for the SSH model, in order to show that the two approaches are equivalent.

The SSH model. The SSH model describes the poly-acetylene chain as a one-dimensional dimerized lattice [5], where each unit cell consists of two sites A and B. Complex amplitudes t and t' quantify the hopping between adjacent sites, belonging to the same unit cell or not, respectively (for the purposes of our analysis it is sufficient to consider t and t' as real parameters). The Hamiltonian describing the electron dynamics along this dimerized lattice is typically introduced for a multi-particle system; accordingly we express it using the second quantization formalism:

$$\hat{H} = \left\{ \sum_{n=1}^{N} t\, \hat{c}^{\dagger}_{A,n} \hat{c}_{B,n} + \sum_{n=1}^{N-1} t'\, \hat{c}^{\dagger}_{A,n+1} \hat{c}_{B,n} \right\} + \text{H.C.}. \tag{3}$$

Here, $\hat{c}^{\dagger}_{A(B),n}$ and $\hat{c}_{A(B),n}$ are the creation and annihilation operators for an electron in the cell n, on the sublattice A (B), and H.C. stands for "Hermitian conjugate". We are considering a finite chain made of N cells, which are spaced by a distance a that we will assume to be equal to one, so as to switch to dimensionless units. Using the Fourier transform for the

creation and annihilation operators

$$\hat{a}_k = \frac{1}{\sqrt{N}} \sum_n e^{-ikn} \hat{c}_{A,n}, \tag{4}$$

$$\hat{b}_k = \frac{1}{\sqrt{N}} \sum_n e^{-ikn} \hat{c}_{B,n} \tag{5}$$

we can express the Hamiltonian operator in momentum space:

$$\hat{H} = \sum_k \hat{\psi}_k^\dagger \{[t + t' \cos(k)]\, \hat{\sigma}_x + t' \sin(k)\, \hat{\sigma}_y\} \hat{\psi}_k. \tag{6}$$

In the latter equation, $\hat{\psi}_k$ is a 2D vector operator defined as

$$\hat{\psi}_k = \begin{pmatrix} \hat{a}_k \\ \hat{b}_k \end{pmatrix}, \tag{7}$$

and $\hat{\sigma}_x$ and $\hat{\sigma}_y$ are the 2D Pauli operators.
The Bloch Hamiltonian is a 2×2 matrix:

$$H(k) = [t + t' \cos(k)]\, \hat{\sigma}_x + t' \sin(k)\, \hat{\sigma}_y \tag{8}$$

Its eigenvalues, representing the system energies for each k, are:

$$E(k) = \pm\sqrt{\left[t^2 + t'^2 - 2tt' \cos(k)\right]} \tag{9}$$

The components of the real 3D unit vector that determines the position of the energy eigenstates on the Bloch (or Poincaré) sphere representing the 2D sublattice Hilbert space are:

$$\begin{aligned} n_x(k) &= \frac{t - t' \cos k}{\sqrt{t^2 + t'^2 - 2tt' \cos k}}, \\ n_y(k) &= -\frac{t' \sin k}{\sqrt{t^2 + t'^2 - 2tt' \cos k}}, \\ n_z(k) &= 0. \end{aligned} \tag{10}$$

We can observe that the z component of $\mathbf{n}(k)$ is vanishing; this results from the absence of $A - A$ and $B - B$ links in the Hamiltonian. Accordingly, the vector $\mathbf{n}(k)$ is confined on

the equator of the Poincaré sphere that we use to represent the 2D sublattice Hilbert space. The two energy bands have a finite gap, vanishing when $t = t'$. Configurations $t < t'$ and $t > t'$ are not equivalent, being the winding number of $\mathbf{n}(k)$, as k varies in the Brillouin zone $\{-\pi, \pi\}$, equal to one and zero, respectively (the winding number is the same topological invariant that we introduced for the QW). In Supplementary Fig. 2 we plot the second order moment of the probability distribution associated with an electron starting localized at a specific lattice site, after a long time evolution. As shown in the figure, this quantity has a slope discontinuity at the transition point $t = t'$, and it is symmetric with respect to an exchange between such parameters, that is $t \leftrightarrow t'$. It is clear that, if the system is driven along any trajectory in the (t, t') space, non-analitycities of the second order moment would reveal if such trajectory crosses the boundary that separates the two phases.

Bardeen-Cooper-Schrieffer(BCS) theory of superconductivity. The effective Hamitonian for a 1D superconductor characterized by p-wave pairing between Cooper pairs can be written in the momentum space as [6]:

$$\hat{H} = \sum_k \left(\frac{\hbar^2 k^2}{2m} - \mu \right) \hat{c}_k^\dagger \hat{c}_k + \Delta k \hat{c}_k \hat{c}_{-k} + \text{H.C.} \tag{11}$$

where m is the electron effective mass, μ is a chemical potential fixing the number of electrons, Δ is the energy gap, $\hat{c}^\dagger_{k(-k)}$ and $\hat{c}_{k(-k)}$ are the creation and annihilation operators for an electron with momentum $k(-k)$, and H.C. stands for "Hermitian conjugate". Electrons with momentum k and $-k$ are coupled together in a Cooper pair, and therefore are created and annihilated in pairs. Each pair of particles is in a relative p-wave (l=1) state. Introducing the 2D vector operator

$$\hat{\psi}_k = \begin{pmatrix} \hat{c}_k \\ \hat{c}^\dagger_{-k} \end{pmatrix}, \tag{12}$$

we can express the Hamiltonian operator in this form:

$$\hat{H} = \sum_k \hat{\psi}_k^\dagger \left\{ \Delta k \, \hat{\sigma}_x + \frac{1}{2} \left(\frac{\hbar^2 k^2}{2m} - \mu \right) \hat{\sigma}_z \right\} \hat{\psi}_k, \tag{13}$$

where $\hat{\sigma}_x$ and $\hat{\sigma}_z$ are the 2D Pauli operators. In order to obtain the lattice effective model,

we replace k and k^2 with $\sin k$ and $4[\sin{(k/2)}]^2$, respectively. The Bloch Hamiltonian is now:

$$\hat{H}(k) = \Delta \sin k \, \hat{\sigma}_x + \frac{\hbar^2}{m}[\sin{(k/2)}]^2 \, \hat{\sigma}_z - \frac{\mu}{2} \, \hat{\sigma}_z. \tag{14}$$

It is straightforward to notice that this Hamiltonian has the same symmetries as the SSH one. The energy eigenvalues are:

$$E(k) = \pm \sqrt{(\Delta \sin k)^2 + \left\{ \frac{\hbar^2}{m}[\sin{(k/2)}]^2 - \frac{\mu}{2} \right\}^2} \tag{15}$$

The real 3D unit vector that determines the position of the energy eigenstates on the Bloch (Poincaré) sphere representing the 2D Hilbert space is:

$$n_x(k) = \frac{\Delta \sin k}{\sqrt{(\Delta \sin k)^2 + \left\{ \frac{\hbar^2}{m}[\sin{(k/2)}]^2 - \frac{\mu}{2} \right\}^2}}, \tag{16}$$

$$n_y(k) = 0,$$

$$n_z(k) = \frac{\frac{\hbar^2}{m}[\sin{(k/2)}]^2 - \frac{\mu}{2}}{\sqrt{(\Delta \sin k)^2 + \left\{ \frac{\hbar^2}{m}[\sin{(k/2)}]^2 - \frac{\mu}{2} \right\}^2}}.$$

We can observe that now the y component of $\mathbf{n}(k)$ is vanishing, therefore the eigenvectors are confined on a great circle of the Bloch sphere contained in the plane perpendicular to the equator. The two energy bands are separated by a finite gap, vanishing when $\mu = 0$. Configurations $\mu < 0$ and $\mu > 0$ are not equivalent, being the winding number of $\mathbf{n}(k)$, as k varies in the Brillouin zone $\{-\pi, \pi\}$, equal to zero and one, respectively. The phase with $\mu > 0$ is the weak pairing phase and the phase with $\mu < 0$ is the strong pairing phase, that is the superconductive phase. As shown in figure Supplementary Fig. 3, even for this system the asymptotic limit of the quantity $\mathcal{M}_2$ for a particle starting localized at a lattice site presents a slope discontinuity at the transition point $\mu = 0$.

The split-step quantum walk. Proposed by Kitagawa et al. [7, 8], the split-step quantum walk is a discrete 1D quantum walk protocol that is defined through the following

unitary evolution operator:

$$\hat{U}_0 = \hat{T}_+ \hat{R}_y(2\theta_2) \hat{T}_- \hat{R}_y(2\theta_1), \tag{17}$$

where $\hat{R}_y(2\theta_1)$ and $\hat{R}_y(2\theta_2)$ are rotations of the coin state about the y axis, and $\hat{T}_+$ and $\hat{T}_-$ are coin dependent shifts of the walker position:

$$\hat{T}_+ = \sum_x |x+1\rangle\langle x| \otimes |+\rangle\langle +| + |x\rangle\langle x| \otimes |-\rangle\langle -| \tag{18}$$

$$\hat{T}_- = \sum_x |x\rangle\langle x| \otimes |+\rangle\langle +| + |x-1\rangle\langle x| \otimes |-\rangle\langle -| \tag{19}$$

The Bloch effective Hamiltonian corresponding to this unitary evolution is a 2x2 matrix acting in the coin space. The effective quasi-energies are $\pm E(k)$, where $E(k)$ is given by the following dispersion relation:

$$\cos E(k) = \cos(\theta_2)\cos(\theta_1)\cos k - \sin(\theta_2)\sin(\theta_1). \tag{20}$$

The real 3D unit vector that determines the position of the effective Hamiltonian eigenstates on the Poincaré sphere representing the coin Hilbert space is:

$$n_x(k) = \frac{\cos(\theta_2)\sin(\theta_1)\sin k}{\sqrt{1-(\cos(\theta_2)\cos(\theta_1)\cos k - \sin(\theta_2)\sin(\theta_2))^2}}, \tag{21}$$

$$n_y(k) = \frac{\cos(\theta_2)\sin(\theta_1)\cos k + \sin(\theta_2)\cos(\theta_1)}{\sqrt{1-(\cos(\theta_2)\cos(\theta_1)\cos k - \sin(\theta_2)\sin(\theta_2))^2}},$$

$$n_z(k) = \frac{-\cos(\theta_2)\cos(\theta_1)\sin k}{\sqrt{1-(\cos(\theta_2)\cos(\theta_1/2)\cos k - \sin(\theta_2)\sin(\theta_2))^2}}$$

Blocking θ_1, as θ_2 varies in the range $\{0, 2\pi\}$ the eigenstates are confined on a great circle of the Poincaré sphere of the coin space. The winding number is one when $|\frac{\tan(\theta_2)}{\tan(\theta_1)}| < 1$ and zero when $|\frac{\tan(\theta_2)}{\tan(\theta_1)}| > 1$; when $|\frac{\tan(\theta_2)}{\tan(\theta_1)}| = 1$ the energy gap closes. Since the effective energy is periodic, the gap can close in $E = 0$ and in $E = -\pi = \pi$.

Here we use the topological classification introduced by Asboth et al. in [9] in terms of

the two invariants Q_0 and Q_π. Following [9], the parameter space (θ_1, θ_2) is divided in four different gapped topological phases, with topological invariants Q_0 and Q_π as shown in Supplementary Fig.4a. An operative way to calculate these invariants is to select a point of the parameter space as a reference and set $(Q_0, Q_\pi) = (0, 0)$; then, for every point in parameter space, we pick a continuous path connecting the latter with such a reference point and count the parity of the number of times the energy gap closes in $E = 0$ and $E = \pi$ along the path. The parities give the values of the invariants Q_0 and Q_π. As shown in Supplementary Fig. 5 every change of the topological numbers (Q_0, Q_π) corresponds to a slope discontinuity of $\mathcal{L}(\theta_1, \theta_2)$, that is the asymptotic value of the second order moment of the probability distribution for a walker which starts localized on a site. Moving in the parameter space along any path which crosses a phase transition, $\mathcal{L}(\theta_1, \theta_2)$ shows discontinuities at the crossing points. Results associated with numerical simulations reported in Supplementary Fig. 5a-b are obtained following the two paths shown in Supplementary Fig.4; here colors are used to associate such numerical results with the corresponding curve in the phase space.

A quantum walk with chiral symmetry only. We analyze now another one-dimensional quantum walk proposed by Kitagawa et al. in his topological classification of QWs [8]. This system has only the sublattice or chiral symmetry. The unitary evolution operator which defines the protocol is:

$$\hat{U}_0 = \hat{T}_+ \hat{R}_\alpha(2\theta_2) \hat{T}_- \hat{R}_\alpha(2\theta_1) \tag{22}$$

where $\hat{R}_\alpha(2\theta_1)$ and $\hat{R}_\alpha(2\theta_2)$ are rotations of the coin state about the direction $\alpha = \frac{1}{\sqrt{2}}(0, 1, 1)$, and $\hat{T}_+$ and $\hat{T}_-$ are the coin-dependent shifts of the walker position defined in Supplementary Eq. 18 and Supplementary Eq. 19. The Bloch effective Hamiltonian corresponding to this unitary evolution is a bidimensional matrix acting in the coin space. The effective energy eigenvalues are $\pm E(k)$, where $E(k)$ is given by the following dispersion relation:

$$\cos E(k) = \frac{1}{2} \sin(\theta_1) \sin(\theta_2) (1 - \cos k) + \cos(\theta_1) \cos(\theta_2) \cos k + \frac{1}{\sqrt{2}} \sin(\theta_1 + \theta_2) \sin k \tag{23}$$

The real 3D unit vector that determines the position of the effective energy eigenstates on

the Poincaré sphere representing the coin Hilbert space is:

$$n_x(k) = \frac{\sin(\theta_1)\left(\sqrt{2}\cos(\theta_2)\sin k + \sin(\theta_2)(1-\cos k)\right)}{\sqrt{1-(\cos E(k))^2}}, \tag{24}$$

$$n_y(k) = \frac{-\sqrt{2}\cos k\cos(\theta_2)\sin(\theta_1) - \sqrt{2}\cos(\theta_1)\sin(\theta_2)}{\sqrt{1-(\cos E(k))^2}} + \\ + \frac{\sin k\sin(\theta_1)\sin(\theta_2)}{\sqrt{1-(\cos E(k))^2}}, \tag{25}$$

$$n_z(k) = \frac{\sin k\cos(\theta_1)\cos(\theta_2) - \frac{1}{\sqrt{2}}\cos k\sin(\theta_2+\theta_1)}{\sqrt{1-(\cos E(k))^2}} + \\ - \frac{\frac{1}{2}\sin k\sin(\theta_1)\sin(\theta_2)}{\sqrt{1-(\cos E(k))^2}} \tag{26}$$

Similarly to the previous case, topological invariants Q_0 and Q_π can be defined for this model; in Supplementary Fig. 4a we plot the associated topological map in terms of the angles θ_1 and θ_2. As shown in Supplementary Fig. 5, the quantity $\mathcal{M}_2$ calculated after many steps starting from $x = 0$ presents slope discontinuities at the transition points.

A quantum walk with particle-hole symmetry only. The last system we consider is a quantum walk protocol that possesses only the particle-hole symmetry. The unitary evolution operator which defines the protocol is:

$$\hat{U}_0 = \hat{T}_+\hat{R}_y(2\theta_2)\hat{T}_-\hat{R}_y(2\theta_1)\hat{T} \tag{27}$$

where $\hat{R}_y(2\theta_1)$ and $\hat{R}_y(2\theta_2)$ are rotations of the coin state about the y axis, $\hat{T}_+$ and $\hat{T}_-$ are the coin-dependent shifts of the walker position defined in Supplementary Eq. 18 and Supplementary Eq. 19, and $\hat{T}$ is the following operator:

$$\hat{T} = \sum_x |x+1\rangle\langle x| \otimes |+\rangle\langle +| + |x-1\rangle\langle x| \otimes |-\rangle\langle -|. \tag{28}$$

The Bloch effective Hamiltonian corresponding to this unitary evolution is a bidimensional matrix acting in the coin space. The effective energy eigenvalues are $\pm E(k)$, where $E(k)$ is

given by the following dispersion relation:

$$\cos E(k) = \cos(\theta_1)\cos(\theta_2)((\cos k)^2 - (\sin k)^2) + \\ - \cos k \sin(\theta_1)\sin(\theta_2). \quad (29)$$

The real 3D unit vector that determines the position of the effective energy eigenstates on the Poincaré sphere representing the coin Hilbert space is:

$$n_x(k) = \frac{\sin(\theta_2)\cos(\theta_1)\sin k}{\sqrt{1-(\cos E(k))^2}}, \quad (30)$$

$$n_y(k) = \frac{-\cos k\cos(\theta_1)\sin(\theta_2) - \cos(\theta_2)\sin(\theta_1)}{\sqrt{1-(\cos E(k))^2}},$$

$$n_z(k) = \frac{2\cos k\sin k\cos(\theta_1)\cos(\theta_2) - \sin k\sin(\theta_2)\sin(\theta_1)}{\sqrt{1-(\cos E(k))^2}}.$$

Since there is no chiral symmetry confining the eigenvectors on a plane, we cannot define a simple winding number. Then, blocking θ_1, the topological phases correspond to two different values of the Berry phase that is still well defined. Q_0 and Q_π are properly defined as well, and their values are reported in Supplementary Fig. 4c. Once again, the quantity $\mathcal{M}_2$ calculated after many steps starting from $x = 0$ presents slope discontinuities at the transition points, see Supplementary Fig.5e-f.

Supplementary Note 3. ANALYSIS OF FIRST AND SECOND ORDER MOMENTS FOR THE SSH MODEL

The approach we illustrated in the previous section can be used to determine the expression of $\mathcal{L}$ for other systems in which Supplementary Eqs. 1-2 are valid. As an example, here we solve analytically integral 2 when the group velocity $\mathcal{V}$ is that of the SSH model. Moreover, we compare this analytical expression with the result obtained from numerical simulation of the SSH dynamics of an electron starting localized at a specific lattice site.

For the SSH model, it can be shown that

$$\mathcal{M}_1/\tau = -s_2\mathcal{L}(t,t') + O(1/\tau), \tag{31}$$

$$\mathcal{M}_2/\tau^2 = \mathcal{L}(t,t') + O(1/\tau^2), \tag{32}$$

We recall that these expressions are obtained when considering an electron prepared in a localized initial state. While this is a standard choice for QWs, in electron dynamics this condition is hard to be reproduced experimentally, and typically is not considered. Even in this case, $\mathcal{L}$ has an analytical expression. Passing to the complex variable $z = \mathrm{e}^{i\,k}$, we find

$$\mathcal{L} = -\frac{i\,tt'}{8\pi}\oint \mathrm{d}z\,\frac{(z^2-1)^2}{z^2\left[z^2 - z\left(\frac{t'^2+t^2}{tt'}\right)+1\right]} \tag{33}$$

where the integral is evaluated along the unit circle $|z| = 1$ in the complex plane. Supplementary Eq. 33 can be solved by residue method. The integrand function has three poles, which are located along the real axis:

$$z_0 = 0; \qquad z_1 = t/t'; \qquad z_2 = t'/t; \tag{34}$$

It is worth noticing that z_0 is doubly degenerate. When varying t and t', poles z_1 and z_2 move in the complex plane, entering or exiting the unit circle. Since only inner poles contribute to the integral (33), residues at z_1 and z_2 should not be considered simultaneously; thus, in the expression of $\mathcal{L}$ the residue at z_1 (z_2) will appear when $t < t'$ ($t > t'$). If $t = t'$, z_1 and z_2 are located on the integration path (the unit circle) and the Supplementary Eq. 33 cannot be solved using this method. The residues at the poles $z : k$ reported in Supplementary Eq. 34 are

$$2\pi i\,r_0 = \frac{t^2+t'^2}{4}; \qquad 2\pi i\,r_1 = \frac{t^2-t'^2}{4}; \qquad 2\pi i\,r_2 = \frac{t'^2-t^2}{4}. \tag{35}$$

Accordingly, we have that

$$\mathcal{L}(t,t') = \begin{cases} 2\pi i(r_0 + r_2) = \frac{t'^2}{2} & \text{for } t' < t \\ \\ 2\pi i(r_0 + r_1) = \frac{t^2}{2} & \text{for } t' > t \end{cases} \tag{36}$$

In Supplementary Fig. 6 we plot the function $\mathcal{L}$, and we compare it to simulated results for the evolution of the electron dynamics described by the SSH model. In the large time limit, numerical data converge rapidly to $\mathcal{L}$ (36). It is important to observe that, at variance with what happens with the QW model, here M_2 would vary in the non-trivial phase too for any variation of the parameter t, that we previously considered as a constant. The observable quantity that is locked to a constant in the non-trivial phase, independently of the hopping parametrization, is M_2/t^2. Remarkably, as for the QW system, this is equal to n_y^2, integrated over the Brillouin zone in momentum space.

---

[1] Ryu, S., Schnyder, A. P., Furusaki, A. and Ludwig, A. W. W. Topological insulators and superconductors: tenfold way and dimensional hierarchy. *New J. of Phys.*, 12:065010 (2010).

[2] Schnyder, A. P., Ryu, S., Furusaki, A. and Ludwig, A. W. W. Classification of topological insulators and superconductors in three spatial dimensions. *Phys. Rev. B*, 78:195125, Nov (2008).

[3] Kitaev, A. Periodic table for topological insulators and superconductors. *AIP Conf. Proc.*, 1134:22 (2009).

[4] Altland, A. and Zirnbauer, M. R. Nonstandard symmetry classes in mesoscopic normal-superconducting hybrid structures. *Phys. Rev. B*, 55:1142–1161 (1997).

[5] Su, W., Schrieffer, J. and Heeger, A. Solitons in polyacetylene. *Phys. Rev. Lett.*, 42:1698–1701 (1979).

[6] Read, N. and Green, D. Paired states of fermions in two dimensions with breaking of parity and time-reversal symmetries and the fractional quantum Hall effect. *Phys. Rev. B*, 61:10267 (2000).

[7] Kitagawa, T. et al. Observation of topologically protected bound states in photonic quantum walks. *Nat. Commun.*, 3(882) (2012).

[8] Kitagawa, T., Rudner, M. S., Berg, E. and Demler, E.. Exploring topological phases with quantum walks. *Phys. Rev. A*, 82:033429 (2010).

[9] Asbóth, J. K. and Obuse, H. Bulk-boundary correspondence for chiral symmetric quantum walks. *Phys. Rev. B*, 88:121406 (2013).
